# Supplementary material for: Brain defence by the extracellular matrix protein Cochlin
Source: Commun Biol. 2026 Sep 16;9:1205. doi: 10.1038/s42003-026-10957-8 (PMC13582957; doi:10.1038/s42003-026-10957-8)
Supplement: Supplementary file 1 [file 42003_2026_10957_MOESM1_ESM.pdf]

nature portfolio

Peer Review File

Brain defence by the extracellular matrix protein Cochlin

Corresponding Author: Dr Suresh Jesuthasan

This file contains all reviewer reports in order by version, followed by all author rebuttals in order by version.

Version 0:

Reviewer comments:

Reviewer #1

(Remarks to the Author)
Ghosh et al., investigated the protective role of Cochlin (Coch), an extracellular matrix anchoring protein expressed at several brain barriers, in host defense against Mycobacterium marinum infection. The authors aimed to demonstrate that coch is evolutionarily conserved across fish, mammals, and birds, and that its expression is localized to brain barrier structures. To support this, they analyzed multiple previously published and publicly available datasets, showing conserved coch expression in the choroid plexus, pineal gland, and selected meningeal structures. In addition to secondary data analysis, the study primarily focused on examining coch expression and function in zebrafish. Using zebrafish larvae, the authors demonstrated that coch contributes to brain protection during M. marinum infection, as loss of coch function resulted in increased bacterial dissemination at embryonic stages. While these findings suggest a potential role for Cochlin in brain barrier–mediated immune defense, the study is limited by broad interpretations and a lack of mechanistic insight. Overall, this work is significant in that it introduces a novel protective component to the brain barrier field; however, further mechanistic analyses are needed to strengthen the conclusions prior to publication.

Major:
- Figure 1 shows coch being expressed primarily on the meningeal structures covering the ventral side of the brain – however detailed zebrafish meningeal reports have recently showed expression of meningeal cell populations covering the entire brain by this developmental stage. To reassure that coch is indeed expressed by meningeal layers and not the most exterior neural structures, the authors should show coch HCR together with a counterstain using markers such as ependymin – a known teleost meningeal glycoprotein.
- The authors suggest coch is expressed by a subset of meningeal fibroblasts, based on their analysis of Daniocell transcriptomic data – this data is available only for embryos up to 5 dpf – and no double staining of coch with any meningeal fibroblast marker is shown by the authors, leaving the statement inconclusive – this could be easily address again by a double stain of coch and a meningeal fibroblast marker.
- Figure 1h and 1j are unclear – beside the ear, these images are not clearly labeled, making it hard to interpret in what structures is coch expressed. These should be replaced by clearer representative images with a brightfield background for easier interpretation. Similarly, Figure 1g, although provides some 3D refence, fails to show clearly, the expression of coch is indeed meningeal. These figures are the basis for some of the author's interpretations about coch and its anchoring to the ECM, expression in barrier structures and their mechanism of action against infection, so they should be addressed directly. In its current state, there is no supportive data for these observations. The authors should either present new data validating these points, or otherwise, elaborate these points in the discussion.
- Upon M. marinum injection into the hindbrain of zebrafish, the authors report seeing a slight increase in the expression of coch after 48 hpi, which is shown in Fig 3 accompanied by an increase of M. marinum in the samples – However, coch expression, not M. marinum, goes down at 72 hpi. The authors do not show whether the injected animals show any other signs of inflammation, cell death, edema or other phenotypes related to infection – this is important, as the decrease in coch expression could be due to the cells expressing coch dying due to the infection. As the authors state in later analysis on Fig 4/5, they remove any sick animals from their final counts, potentially missing some of these phenotypes.
- The authors describe the expression of coch in several tissues such as pineal gland, meninges and choroid plexuses, and even describe transcriptomic and imaging data sets from other publications/resources showing that coch is also conserved between birds, mammals and fish, however, they do not address at all – in their results or discussion – whether coch mutants in mammals have shown any phenotypes or sensitivity to infection. The results they show using zebrafish coch mutants are promising and show a potential role for coch in the control of M. marinum infections, however they focus on the meninges as a tissue of interest, but choose timepoints where the meningeal structures are not yet present or formed completely. Previous studies - PMID: 40321204, have described the meninges as forming structures that begin appearing at

around (3dpf) the time points the authors perform their infection and rescue analysis, which would suggest that coch is not acting at the meningeal level in these particular experiments. It would be much more informative to repeat these studies using slightly older larvae/adults where structures that express coch are properly formed.
- The authors also fail to describe any phenotypes that the coch CRISPR mutants may present based on their earlier expression data. Do these mutants have a properly formed/functional pineal gland? Adult choroid plexuses? etc.

Minor:
- The authors refer to the zebrafish as a primitive meninx – this term although classically used, does not align with the most recent publications suggesting that the zebrafish has multilayered meninges, comparable to the mammalian structures, so the authors should consider to simply use the term 'meninges'.
- In methods, the author mention several zebrafish transgenic lines that were not used/described in the figures – e.g. Tg(−5.2lyve1b:EGFP)nz151, among others – also, the authors state in the methods that sex of animals used was not a factor in their analysis – although this is true for embryonic samples, it does not apply to adults, and should be described appropriately.
- The authors use publicly available data set such as the Allen Brain atlas to show the expression of Coch in different brain barrier compartments. It would make the data stronger if they would have shown by HCR or protein staining this expression on their own samples, as this would present a stronger validation of the expression of this particular gene/protein at key time points.

Reviewer #2

(Remarks to the Author)
This is an interesting report aiming to demonstrate secretion of dormant matrix-associated proteins as an important aspect in brain-defense. These propositions are novel and will be of interest to wider field.
Authors should revise a lot of phrases throughout the manuscript text. In abstract they write, "we show that Cochlin, a collagen-binding protein that is cleaved during infection to release". The collagen-binding of Cochlin has not been demonstrated in a direct experimental assay in all tissues or all neuronal tissues. The direct binding that has been demonstrated (for example, PMID: 29305555) is with collagen VII and speculated to extrapolate to collagen IV. In other experiments, for example, in specific ocular or ear tissues these interactions could not be unequivocally demonstrated. So it perhaps better not to refer to Cochlin as a universal collagen-binding protein.
Authors write (pdf page 2), "the pineal gland – releases exosomes to the ventricle (3); given their ability to transport macromolecules, this provides a potential route for disease spread". Authors should break down these sentences to add clarity. Exosomes are outwardly secreted entity. Unlike lysosomes or phagosomes exosomes are not considered to be part of degradation compartments. So the descriptions of potential route of disease spread in the context of communicable diseases should be re considered and re-phrased. 13). On pdf page 2, the description is, "Cochlin is anchored to the extracellular matrix (ECM) via two von Willebrand domains (14)" ECM in most tissues around cells are a fleeting component. Authors should consider consulting following paper as to how ECM changes over time course (PMID: 26299746) organ-specific ECM interactions (PMID: 33045682). Anchored to the ECM is not an appropriate description. Authors can easily rephrase it. On pdf page 5, authors mention, "Unexpectedly, Coch was also detected in the third and lateral ventricle choroid plexus of adult mice (Fig. S7d-I, S8d,e) (20–23)." why this is unexpected? Readership will be at a loss. Authors should clarify why it is unexpected. On page 6, "2i,l). Additionally, meningeal expression was detected in a discrete band at the boundary of the brain and spinal cord in 14 dpf and 30 dpf fish (Fig. 2i-n). This potentially represents the meninges around the brainstem at the foramen magnum of the zebrafish skull." Authors should add clarity, solid experimental evidence or remove the description "potentially represents".

Authors should consider citation of the following relevant older work: PMID: 16951386, PMID: 15085200. Authors have used mycobacterium. However, Porphyromonas gingivalis and Pseudomonas are also common brain external pathogen in zebrafish. For murine they mention gram positive and gram negative both. Authors should consider some incorporation of data for pathogen other than mycobacterium. meninges. A big part of their novel conclusion is, "Cochlin is likely secreted into the ventricle from the circumventricular organs and choroid plexus, where it anchors to the matrix. This enables Cochlin to act on bacteria outside the blood-brain barrier, complementing its previously reported function in the spleen (10)." However, this conclusion is marred by lack of evidence for Cochlin secretion. In many placed authors use the phrase "potential" and here "likely". For wider audience they should present some broad based evidence for at least one more pathogen and demonstrate some secretion. Why accumulation of Cochlin have been found in some other brain location is also speculative. It is alright to have some aspects described as potential and likely but what is part of main conclusions should be solidified with some experimental data.

Version 1:

Reviewer comments:

Reviewer #1

(Remarks to the Author)
I think the authors have addressed my major concerns and revised the manuscript to better reflect their interpretation of the data. Overall, these revisions improve the clarity of the manuscript and reduce the potential for misinterpretation of the findings.

As noted in their rebuttal, some of the suggested revisions cannot be completed at this time due to their relocation. In my view, the absence of these data does not constitute a major barrier to publication, but I will defer to the Editor’s judgment on this point.

Reviewer #2

(Remarks to the Author)
Authors have attempted to address all comments to the extent possible. Some comments have been addressed by changing the text, as authors note, due to lab relocation from Singapore to Sweden performing some experiments in timely fashion is not feasible. Under these conditions the best is to modify the text which the authors have done.

Open Access This Peer Review File is licensed under a Creative Commons Attribution 4.0 International License, which permits use, sharing, adaptation, distribution and reproduction in any medium or format, as long as you give appropriate credit to the original author(s) and the source, provide a link to the Creative Commons license, and indicate if changes were made.
In cases where reviewers are anonymous, credit should be given to 'Anonymous Referee' and the source.
The images or other third party material in this Peer Review File are included in the article’s Creative Commons license, unless indicated otherwise in a credit line to the material. If material is not included in the article’s Creative Commons license and your intended use is not permitted by statutory regulation or exceeds the permitted use, you will need to obtain permission directly from the copyright holder.
To view a copy of this license, visit https://creativecommons.org/licenses/by/4.0/

We thank the reviewers for their comments and suggestions on improving the manuscript. Below are detailed responses to each point.

Reviewer #1 (Remarks to the Author):

Ghosh et al., investigated the protective role of Cochlin (Coch), an extracellular matrix anchoring protein expressed at several brain barriers, in host defense against Mycobacterium marinum infection. The authors aimed to demonstrate that coch is evolutionarily conserved across fish, mammals, and birds, and that its expression is localized to brain barrier structures. To support this, they analyzed multiple previously published and publicly available datasets, showing conserved coch expression in the choroid plexus, pineal gland, and selected meningeal structures. In addition to secondary data analysis, the study primarily focused on examining coch expression and function in zebrafish. Using zebrafish larvae, the authors demonstrated that coch contributes to brain protection during M. marinum infection, as loss of coch function resulted in increased bacterial dissemination at embryonic stages. While these findings suggest a potential role for Cochlin in brain barrier–mediated immune defense, the study is limited by broad interpretations and a lack of mechanistic insight. Overall, this work is significant in that it introduces a novel protective component to the brain barrier field; however, further mechanistic analyses are needed to strengthen the conclusions prior to publication.

Major:

- Figure 1 shows coch being expressed primarily on the meningeal structures covering the ventral side of the brain – however detailed zebrafish meningeal reports have recently showed expression of meningeal cell populations covering the entire brain by this developmental stage. To reassure that coch is indeed expressed by meningeal layers and not the most exterior neural structures, the authors should show coch HCR together with a counterstain using markers such as ependymin – a known teleost meningeal glycoprotein.

We have carried out double HCR for *cochlin* and *ependymin* in larval zebrafish, as shown in the revised Fig. 1g, h and Fig. S4. *Ependymin* is expressed in the leptomeninges (Galanternik et al., 2025), i.e. the layer of the meninges that contacts the brain. *Cochlin* is expressed outside this layer. This establishes that *cochlin* is not expressed in a neural structure.

- The authors suggest coch is expressed by a subset of meningeal fibroblasts, based on their analysis of Daniocell transcriptomic data – this data is available only for embryos up to 5 dpf – and no double staining of coch with any meningeal fibroblast marker is shown by the authors, leaving the statement inconclusive – this could be easily address again by a double stain of coch and a meningeal fibroblast marker.

To further characterise cochlin expression in the meninges, we have examined a publicly available single cell transcriptomic dataset obtained from the meninges of adult zebrafish (Galanternik et al., 2025). This indicates that *cochlin* is present in the fibroblast cluster in the pachymeninges, with one marker of this cluster being *pdgfrb*. We have performed HCR for *cochlin* in a transgenic line with eGFP driven by *pdgfrb* enhancers, *TgBAC(pdgfrb:eGFP)* (Ando et al., 2016). As shown in the revised Figure 1i-l, *coch* is expressed in EGFP expressing cells, suggesting co-expression with *pdgfrb.*

- Figure 1h and 1j are unclear – beside the ear, these images are not clearly labeled, making it hard to interpret in what structures is coch expressed. These should be replaced by clearer representative images with a brightfield background for easier interpretation.

We have replaced the images. The new images contain either a nucleic acid stain (Fig. 1n) or a brightfield channel (Fig. 1o), both of which show the larvae. We have also provided additional labels, e.g. for the olfactory epithelium (oe) and optic tectum (ot).

Similarly, Figure 1g, although provides some 3D refence, fails to show clearly, the expression of coch is indeed meningeal. These figures are the basis for some of the author's interpretations about coch and its anchoring to the ECM, expression in barrier structures and their mechanism of action against infection, so they should be addressed directly. In its current state, there is no supportive data for these observations. The authors should either present new data validating these points, or otherwise, elaborate these points in the discussion.

We have performed HCR for *igfbp2a* and *ependymin* (Fig. S4). This indicates that *coch* is expressed adjacent and lateral to the leptomeninges.

- Upon M. marinum injection into the hindbrain of zebrafish, the authors report seeing a slight increase in the expression of coch after 48 hpi, which is shown in Fig 3 accompanied by an increase of M. marinum in the samples – However, coch expression, not M. marinum, goes down at 72 hpi. The authors do not show whether the injected animals show any other signs of inflammation, cell death, edema or other phenotypes related to infection – this is important, as the decrease in coch expression could be due to the cells expressing coch dying due to the infection. As the authors state in later analysis on Fig 4/5, they remove any sick animals from their final counts, potentially missing some of these phenotypes.

The reviewer is correct, as the original analysis did not account for any changes in the number of cells, and reported the sum of the intensity. This was an error, as any reduction could simply be caused by cell death.

In reviewing the data, we have realised that there is a technical problem with this experiment – specifically the samples lacked a second probe that would enable normalisation across different samples (qHCR; Choi et al., 2018). Without this, the analysis lacks robustness. We have thus removed this data.

The authors describe the expression of coch in several tissues such as pineal gland, meninges and choroid plexuses, and even describe transcriptomic and imaging data sets from other publications/resources showing that coch is also conserved between birds, mammals and fish, however, they do not address at all – in their results or discussion – whether coch mutants in mammals have shown any phenotypes or sensitivity to infection.

Coch mutants in mice have infection-induced hearing loss, reflecting expression in the ear, and increased susceptibility to lung infection, reflecting the expression in the spleen. This is noted in lines 168 - 170.

The results they show using zebrafish coch mutants are promising and show a potential role for coch in the control of M. marinum infections, however they focus on the meninges as a tissue of interest, but choose timepoints where the meningeal structures are not yet present or formed completely. Previous studies - PMID: 40321204, have described the meninges as forming structures that begin appearing at around (3dpf) the time points the authors perform their infection and rescue analysis, which would suggest that coch is not acting at the meningeal level in these particular experiments. It would be much more informative to repeat these studies using slightly older larvae/adults where structures that express coch are properly formed.

The manuscript reports *cochlin* expression in a number of barrier tissues, including the pineal gland (Figure 1a-f), area postrema (Fig. 1g, Fig. S2a, e) and choroid plexus (Fig. 2). The meninges is one of the tissues showing expression but is not intended to be the sole focus.

During the period of infection, i.e. 3-7 dpf, *coch* is expressed in the pineal gland and the area postrema, as well as the meninges. The time of bacteria delivery aligns with the start of expression, and there is clear expression while the infection is in progress.

The bacteria were delivered to the ventricle, rather than peripherally. One reason for this is the evidence that mycobacteria can penetrate into the brain.

We agree that repeating the experiments at older animals would be informative, especially as expression in the choroid plexus is seen only at lates stages. Unfortunately, this is not possible, because the lab has relocated from Singapore to Sweden. The ethical approvals in Singapore have lapsed, and the room for performing infection experiments in the Singapore lab is no longer operational. We do not have facilities or ethical approval for infection in Sweden, nor do we have the mutant line with us at present – this could not be imported previously due to regulatory restrictions.

- The authors also fail to describe any phenotypes that the coch CRISPR mutants may present based on their earlier expression data. Do these mutants have a properly formed/functional pineal gland? Adult choroid plexuses? etc.

We have not observed morphological abnormalities in the pineal gland, and did not analyse the adult choroid plexus. Although this would be interesting, it is unfortunately not possible for us to check at present, as the lab has relocated and has not been able to import the mutant line to Sweden yet. We would not be able to carry out this analysis within a reasonable time frame.

Minor:

- The authors refer to the zebrafish as a primitive meninx – this term although classically used, does not align with the most recent publications suggesting that the zebrafish has multilayered meninges, comparable to the mammalian structures, so the authors should consider to simply use the term 'meninges'.

The manuscript has been edited to reflect this change.

- In methods, the author mention several zebrafish transgenic lines that were not used/described in the figures – e.g. Tg(−5.2lyve1b:EGFP)nz151, among others – also, the authors state in the methods that sex of animals used was not a factor in their analysis – although this is true for embryonic samples, it does not apply to adults, and should be described appropriately.

We have removed the description of these lines, as they are not used in the results presented here.

The sex of adult animals used for imaging are indicated in Table S1.

- The authors use publicly available data set such as the Allen Brain atlas to show the expression of Coch in different brain barrier compartments. It would make the data stronger if they would have shown by HCR or protein staining this expression on their own samples, as this would present a stronger validation of the expression of this particular gene/protein at key time points.

We have carried out antibody labelling on the mouse brain. This indicates that Cochlin protein is present in the surface of the meninges, within the choroid plexus and on the ventricle wall. These data are shown in the revised Figure 3.

Reviewer #2 (Remarks to the Author):

This is an interesting report aiming to demonstrate secretion of dormant matrix-associated proteins as an important aspect in brain-defense. These propositions are novel and will be of interest to wider field.
Authors should revise a lot of phrases throughout the manuscript text. In abstract they write, "we show that Cochlin, a collagen-binding protein that is cleaved during infection to release". The collagen-binding of Cochlin has not been demonstrated in a direct experimental assay in all tissues or all neuronal tissues. The direct binding that has been demonstrated (for example, PMID: 29305555) is with collagen VII and speculated to extrapolate to collagen IV. In other experiments, for example, in specific ocular or ear tissues these interactions could not be unequivocally demonstrated. So it perhaps better not to refer to Cochlin as a universal collagen-binding protein.

The text has been edited to state that Cochlin can bind to the extracellular matrix. The evidence for binding specifically to collagen VII is noted on page 2, line 44.

Authors write (pdf page 2), "the pineal gland – releases exosomes to the ventricle (3); given their ability to transport macromolecules, this provides a potential route for disease spread". Authors should break down these sentences to add clarity. Exosomes are outwardly secreted entity. Unlike lysosomes or phagosomes exosomes are not considered to be part of degradation compartments. So the descriptions of potential route of disease spread in the context of communicable diseases should be re considered and re-phrased.

This sentence has been re-written.

13). On pdf page 2, the description is, "Cochlin is anchored to the extracellular matrix (ECM) via two von Willebrand domains (14)" ECM in most tissues around cells are a fleeting component. Authors should consider consulting following paper as to how ECM changes over time course (PMID: 26299746) organ-specific ECM interactions (PMID: 33045682). Anchored to the ECM is not an appropriate description. Authors can easily rephrase it.

The word “binds” is used instead.

On pdf page 5, authors mention, "Unexpectedly, Coch was also detected in the third and lateral ventricle choroid plexus of adult mice (Fig. S7d-I, S8d,e) (20–23)." why this is unexpected? Readership will be at a loss. Authors should clarify why it is unexpected.

The sentence has been rephrased. The observation was initially not expected, as it occurred prior to the observations in zebrafish, but we agree that this is not relevant to the readers.

On page 6, "2i,l). Additionally, meningeal expression was detected in a discrete band at the boundary of the brain and spinal cord in 14 dpf and 30 dpf fish (Fig. 2i-n). This potentially represents the meninges around the brainstem at the foramen magnum of the zebrafish skull." Authors should add clarity, solid experimental evidence or remove the description "potentially represents".

This phrase has been removed.

Authors should consider citation of the following relevant older work: PMID: 16951386, PMID: 15085200.

These papers report the role of pro-inflammatory T-cells that recognise Cochlin in inducing hearing loss. We have added these references to the discussion of the implications of the presence of Cochlin in barrier tissues (lines 304 – 308).

Authors have used mycobacterium. However, Porphyromonas gingivalis and Pseudomonas are also common brain external pathogen in zebrafish. For murine they mention gram positive and gram negative both. Authors should consider some incorporation of data for pathogen other than mycobacterium. meninges.

We agree that the manuscript would be strengthened by the inclusion different bacteria. However, this is difficult to do at present. The lab has recently moved from Singapore to Sweden, and there are no facilities in the facility in Umeå to carry out infection experiments on zebrafish. We also do not have the necessary ethical approval here, or in Singapore, to conduct experiments with other pathogens. We have noted in the discussion (lines 301 – 303) that it would be important to test the effects of Cochlin on other pathogens.

A big part of their novel conclusion is, "Cochlin is likely secreted into the ventricle from the circumventricular organs and choroid plexus, where it anchors to the matrix. This enables Cochlin to act on bacteria outside the blood-brain barrier, complementing its previously reported function in the spleen (10)." However, this conclusion is marred by lack of evidence for Cochlin secretion. In many placed authors use the phrase "potential" and here "likely". For wider audience they should present some broad based evidence for at least one more pathogen and demonstrate some secretion. Why accumulation of Cochlin have been found in some other brain location is also speculative. It is alright to have some aspects described as potential and likely but what is part of main conclusions should be solidified with some experimental data.

We have provided new evidence for secretion of Cochlin into the CSF. Firstly, we have added proteomic data from CSF of adult zebrafish, which shows that Cochlin is present in the CSF. This is documented in the revised Fig.2i. Secondly, we have carried out antibody labelling in the mouse brain, and detected Cochlin protein on the ventricle wall and on the meninges. This is shown in Figure 3. Finally, we note in line 286 that COCH was recently detected in human CSF.

References

Ando, K., Fukuhara, S., Izumi, N., Nakajima, H., Fukui, H., Kelsh, R. N. and Mochizuki, N. (2016). Clarification of mural cell coverage of vascular endothelial cells by live imaging of zebrafish. *Development* **143**, 1328–1339.

Choi, H. M. T., Schwarzkopf, M., Fornace, M. E., Acharya, A., Artavanis, G., Stegmaier, J., Cunha, A. and Pierce, N. A. (2018). Third-generation in situ hybridization chain reaction: multiplexed, quantitative, sensitive, versatile, robust. *Development* **145**, dev165753.

Galanternik, M. V., Castranova, D., Gober, R. D., Nguyen, T., Kenton, M., Margolin, G., Kraus, A., Sur, A., Dye, L. E., Pham, V., et al. (2025). Anatomical and Molecular Characterization of the Zebrafish Meninges. *bioRxiv* 2025.04.09.646894.

Reviewers' comments:

Reviewer #1 (Remarks to the Author):

I think the authors have addressed my major concerns and revised the manuscript to better reflect their interpretation of the data. Overall, these revisions improve the clarity of the manuscript and reduce the potential for misinterpretation of the findings.

As noted in their rebuttal, some of the suggested revisions cannot be completed at this time due to their relocation. In my view, the absence of these data does not constitute a major barrier to publication, but I will defer to the Editor’s judgment on this point.

Response:
We thank the reviewer for their comments. We note that there are no remaining concerns.

Reviewer #2 (Remarks to the Author):

Authors have attempted to address all comments to the extent possible. Some comments have been addressed by changing the text, as authors note, due to lab relocation from Singapore to Sweden performing some experiments in timely fashion is not feasible. Under these conditions the best is to modify the text which the authors have done.

Response:
We thank the reviewer for their comments. We note that there are no remaining concerns.
