# Supplementary material for: Brain defence by the extracellular matrix protein Cochlin
Source: Commun Biol. 2026 Sep 16;9:1205. doi: 10.1038/s42003-026-10957-8 (PMC13582957; doi:10.1038/s42003-026-10957-8)
Supplement: Supplementary file 2 [file 42003_2026_10957_MOESM2_ESM.pdf]

Supplementary Figures

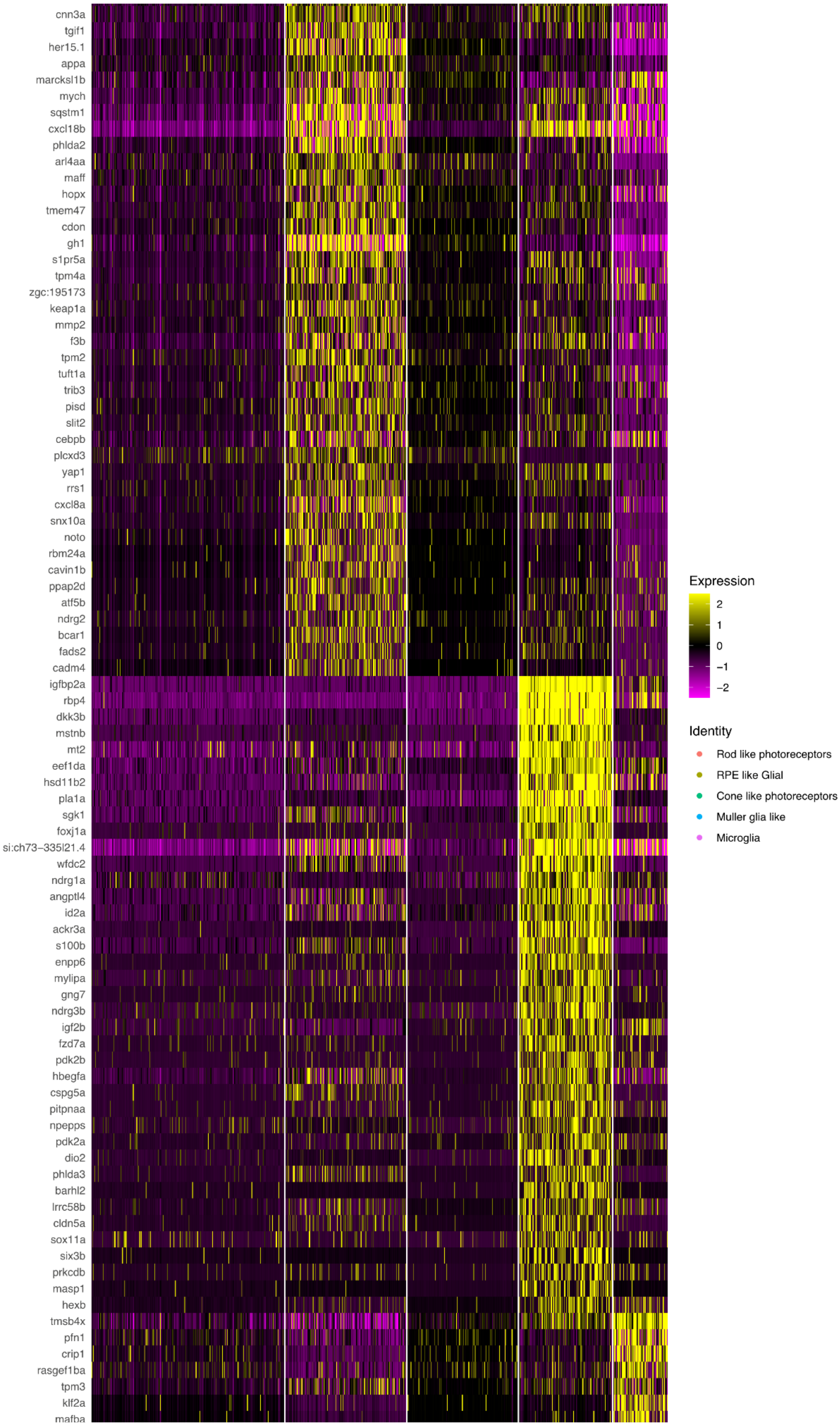

Figure S1. Expression of immune genes in the zebrafish pineal gland. Heatmap of immune related genes filtered from the cluster markers of the adult zebrafish pineal gland (1).

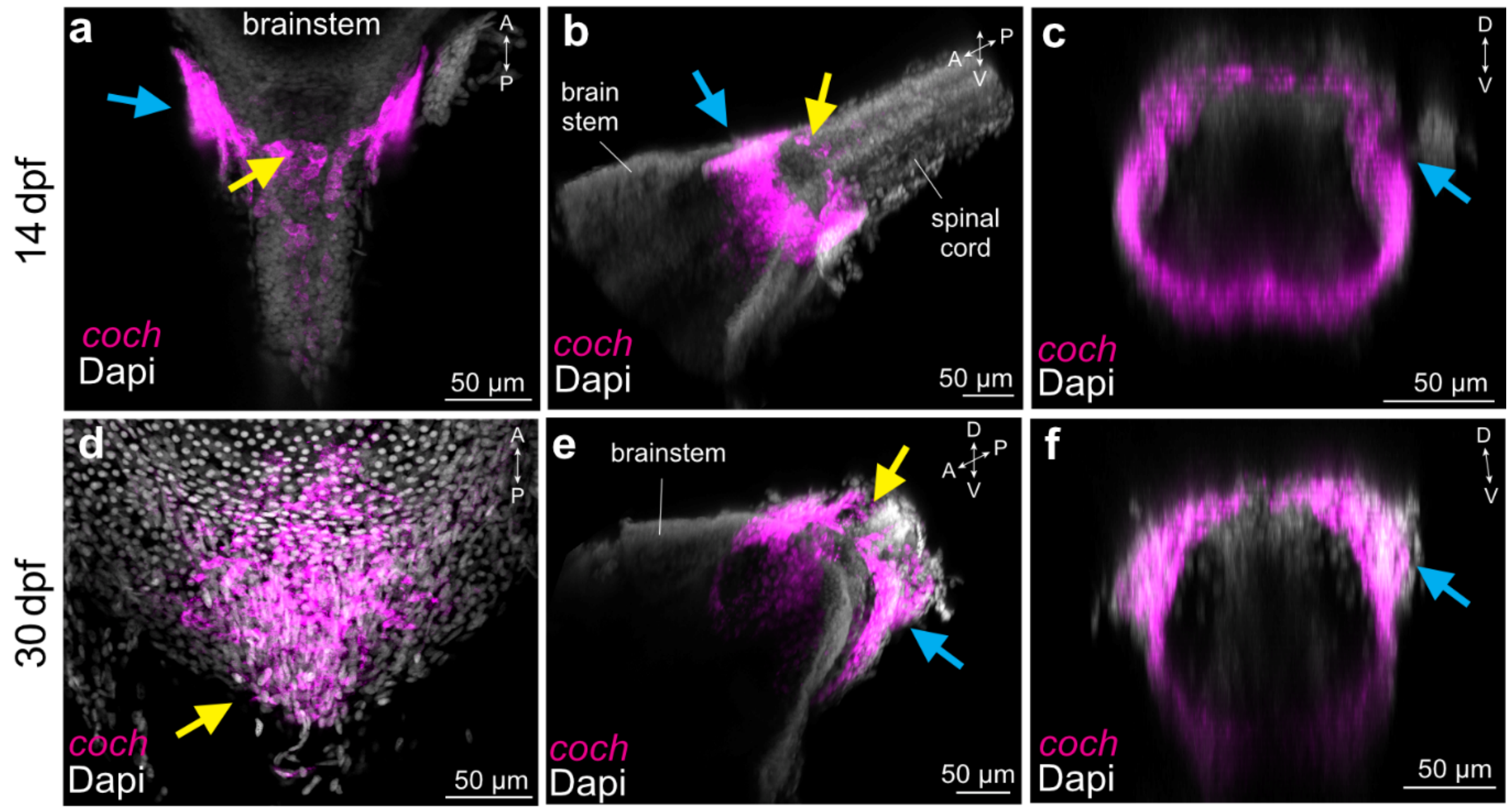

Figure S2. *coch* expression in the area postrema and around the posterior brainstem of zebrafish of 14 and 30 dpf . a-f. HCR-ISH of *coch* mRNA in the brainstem region at (a-c) 14 dpf and (d-f) 30 dpf. *Coch*: magenta, DAPI: grayscale. Yellow dotted lines: basal sides of the choroid plexus epithelial cells that stromal cells are located. Yellow arrows: meningeal expression of the posterior part of the area postrema. Blue arrows: meningeal expression of a discrete band at the boundary of the brain and spinal cord. **a,d.** Dorsal views of max projection images. **b,e**. 3D reconstructed images of the brainstem region. **c,f**. Cross sectional view of the band-like expression in (b, e). A: anterior, P: posterior, D: dorsal, V: ventral.D/MV: Diencephalic/mesencephalic ventricle.

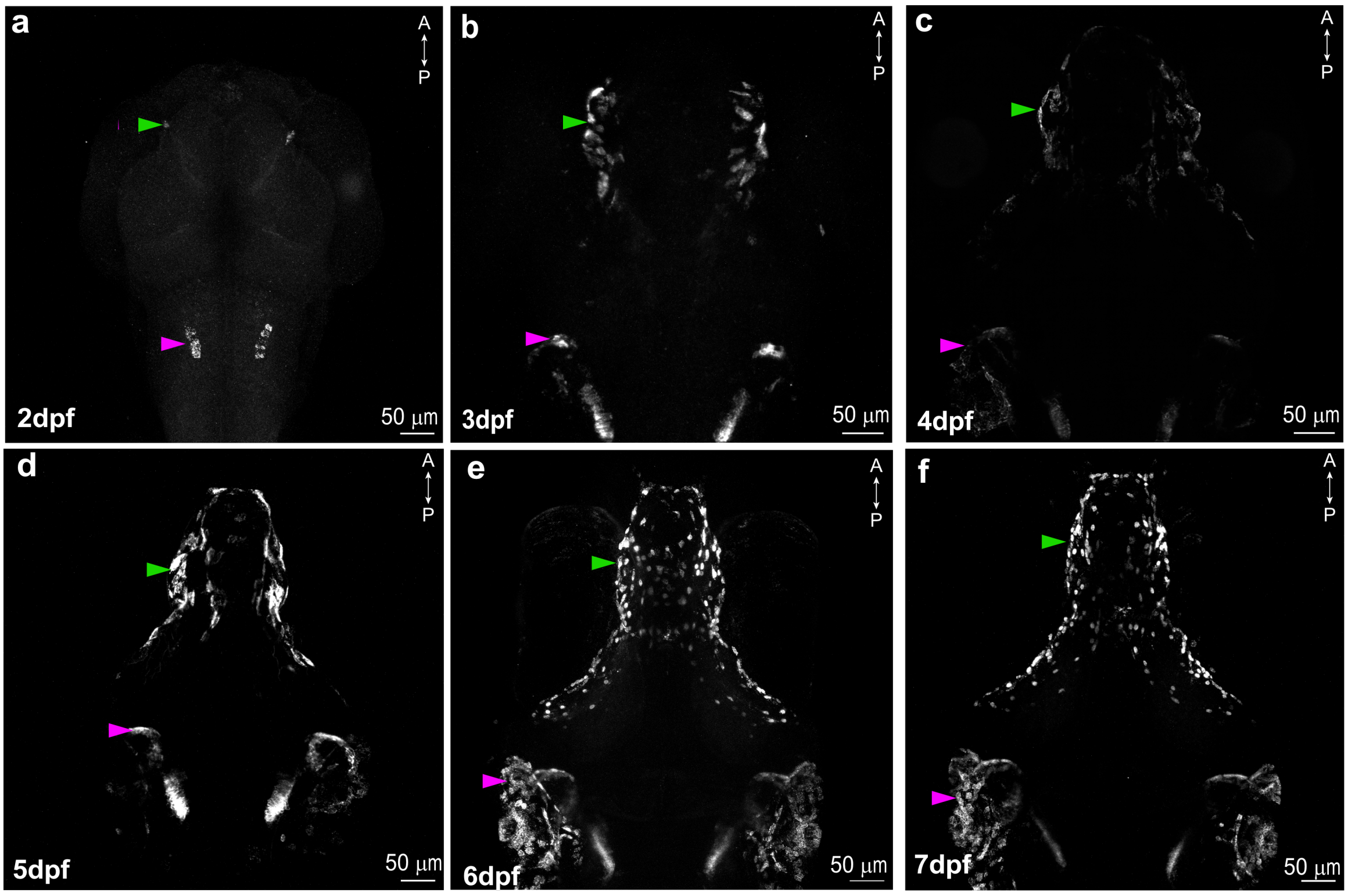

Figure S3. Time series of *coch* expression in the fore- and midbrain of zebrafish larvae up to 7 dpf. **a.** 2 dpf, **b.** 3 dpf, **c.** 4 dpf **d.** 5 dpf, **e.** 6 dpf and **f.** 7 dpf. Arrow heads: green – meningeal expression, magenta – otic expression. Expressing cells are located laterally, and then ventrally to the brain.

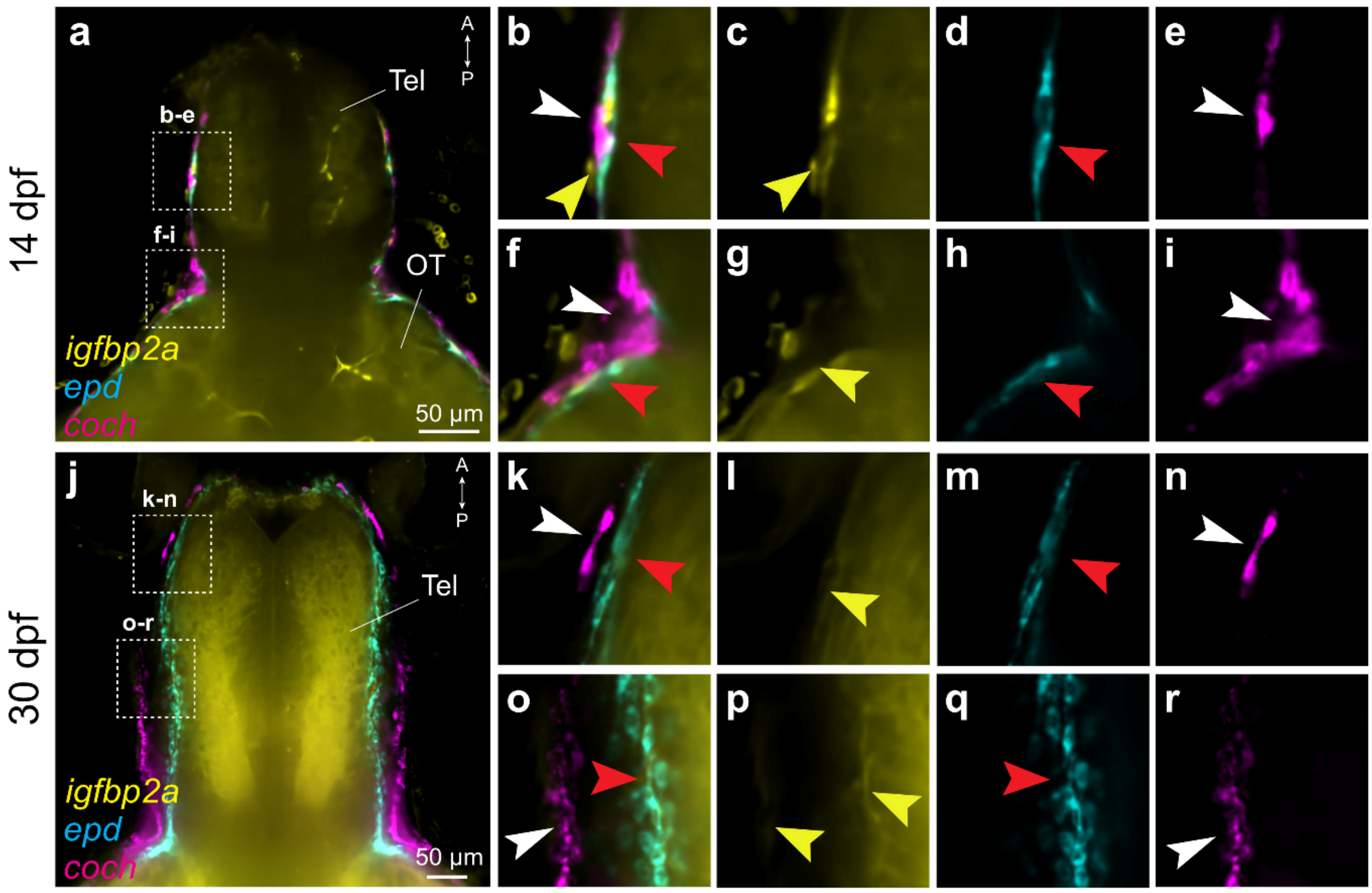

Figure S4. Meningeal *coch* expression with meningeal markers in the meningeal areas of the zebrafish forebrain in 14 and 30 day old zebrafish larvae. **a,j.** Multiplexed HCR-ISH of *coch, epd* and *igfbp2a* mRNAs in (a) 14 dpf and (j) 30 dpf. Dorsal views of single plane images. *coch*: magenta, *igfbp2a*: yellow, *epd*: cyan. Merged channels. White dotted boxes show the location of insets b-e, f-i, k-n and o-r. Tel: Telencephalon, OT: optic tectum. **b-e, f-i**. High magnification images of the white dotted boxes in (a). Merged (b,f) and individual (c&g, d&h and e&i) channels. **k-n, o-r**. High magnification images of the white dotted boxes in (j). Merged (k,o) and individual (l&p, m&q and n&r) channels. White arrowheads: the *coch* positive meningeal cell layer, yellow arrowheads: the *igfbp2a* positive meningeal layer, red arrowheads: the *epd* positive meningeal layer. A: anterior, P: posterior, Tel: telencephalon, OT: optic tectum.

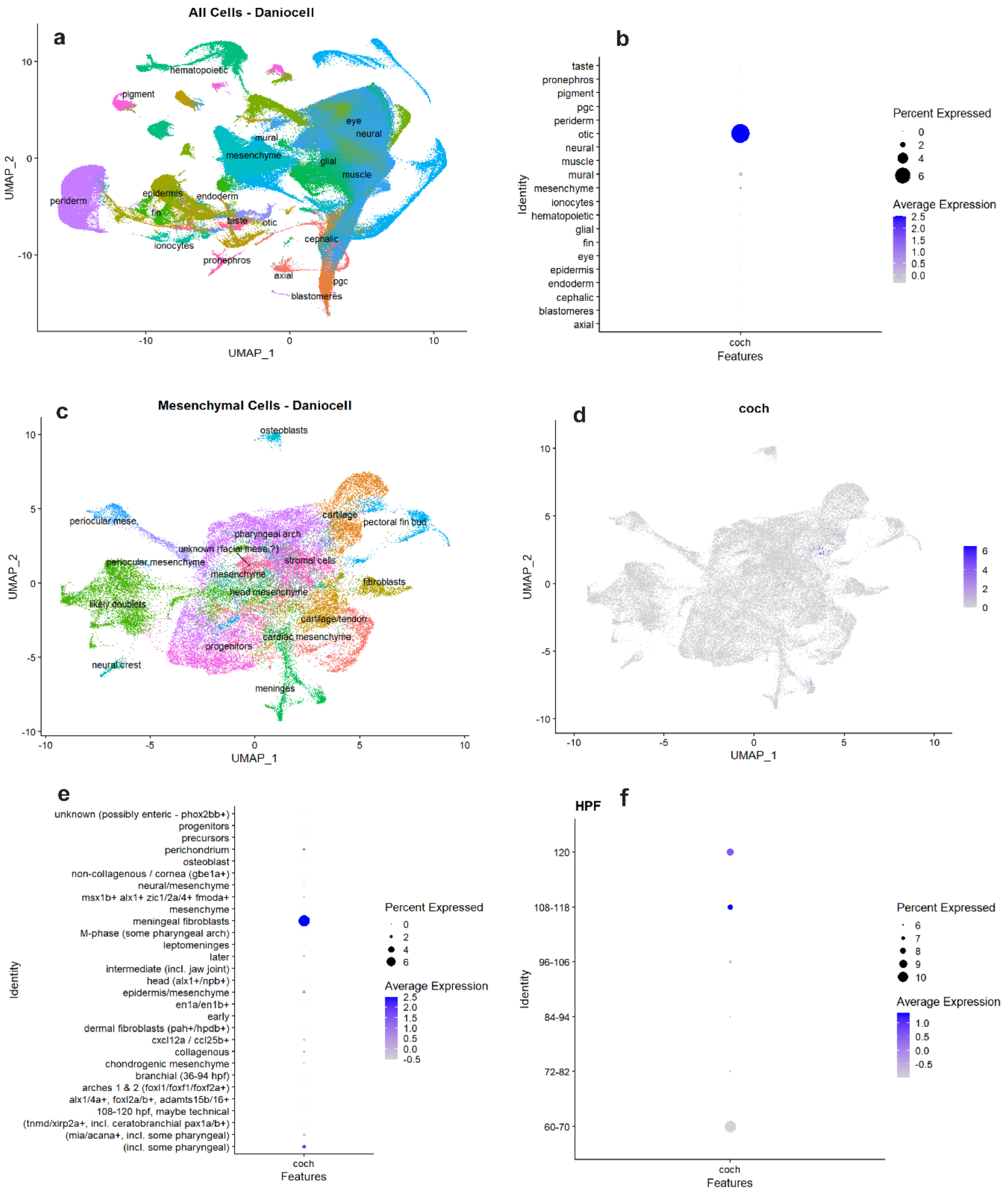

Figure S5. Identification of cells expressing *coch* in the meninges of larval zebrafish. a. UMAP of the Daniocell dataset containing all cells. **b.** Dot plot for *coch* expression within the Daniocell dataset showing highest expression in the otic tissue followed by mural and mesenchymal tissue subsets. **c.** UMAP of mesenchymal tissue cell which have subsetted out of Daniocell dataset. **d.** Feature plot for *coch* expression within the mesenchymal tissue subset showing expression in cartilage and meninges subgroup of mesenchymal cell. **e.** Dotplot for *coch* expression within the mesenchymal tissue subset showing highest

expression within the meningeal fibroblast cluster. **f.** Dotplot for *coch* expression within the meningeal fibroblasts split by hours post fertilization (hpf) stages.

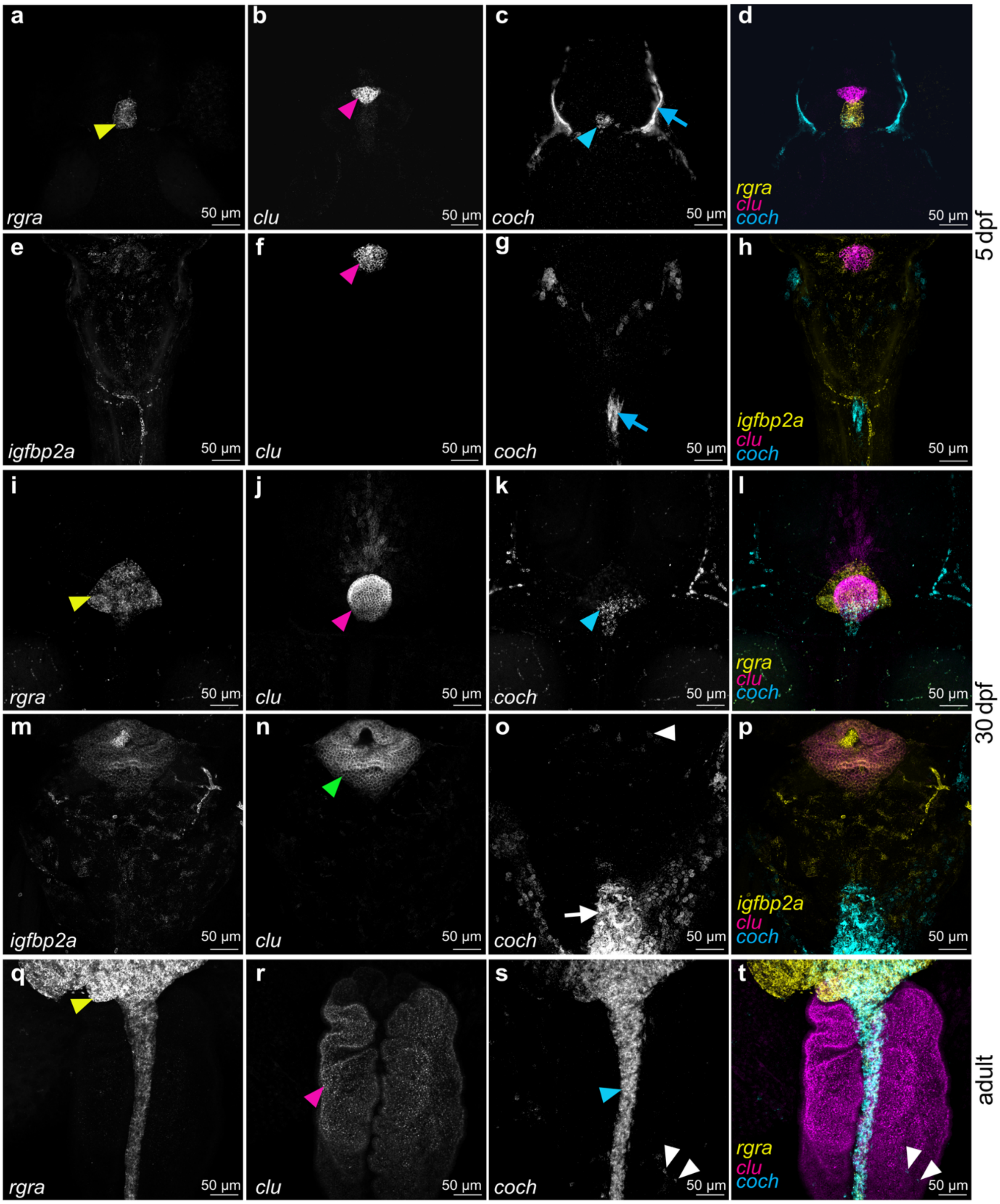

Fig. S6. Coch expression in the choroid plexus. a-t. Multiplexed HCR-ISH of *coch*, *igfbp2a*, *clu* and *retinal G protein coupled receptor a (rgra)* mRNAs in **(a-h)** 5 dpf, **(i-p)** 30 dpf and **(q-t)**

adult fish. All images are maximum projections of z-stacks, showing dorsal views. Expression of **(a, i, q)** *rgra* (yellow arrowheads: the pineal gland), **(e, m)** *igfbp2a* **(b, f, j, n, r)**, *clu* (magenta arrowheads: forebrain choroid plexus epithelial cells, green arrowheads: hindbrain choroid plexus epithelial cells), **(c, g, k, o, s)** *coch* (blue arrowheads: pineal gland, white arrowheads: choroid plexus stroma, blue arrows: forebrain meninges, white arrows: area postrema. **d, h, l, p, t.** merged images of each channel. A: Anterior, P: Posterior. Gamma of panels g, k, o and s has been set to 0.45 for better visualization.

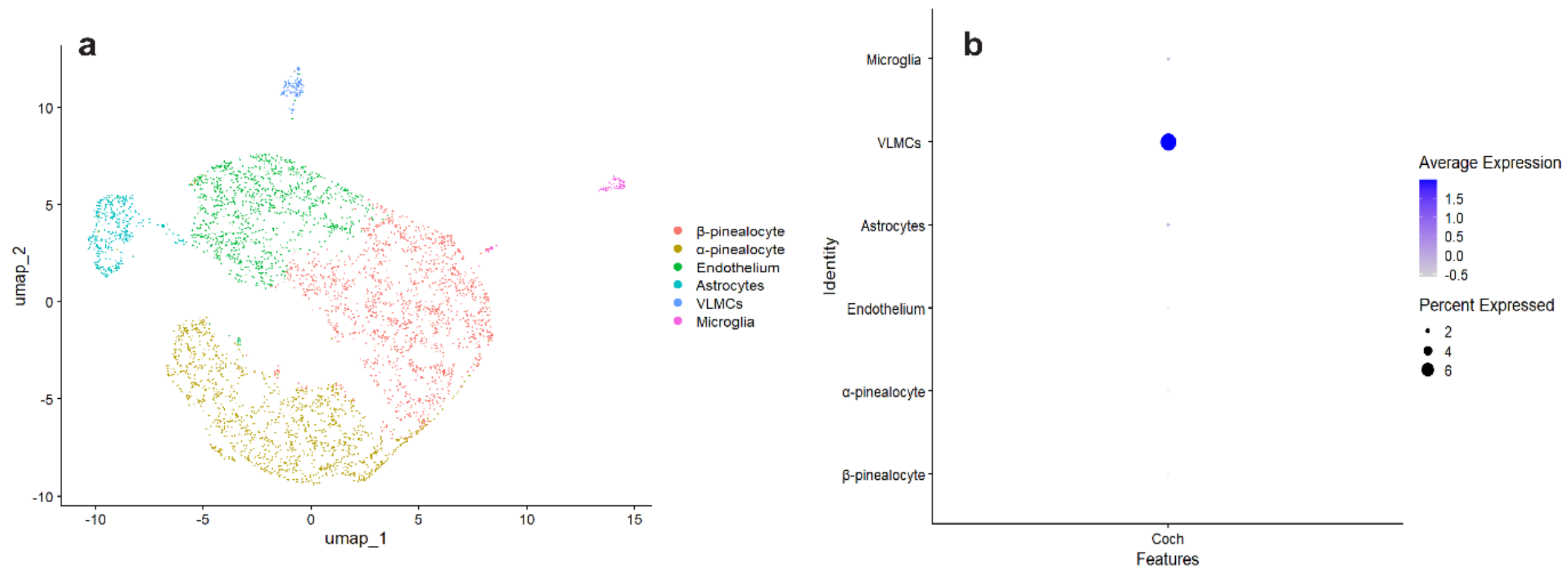

Figure S7. *Cochlin* expression in the rat pineal gland. a. UMAP of integrated day time datasets of rat pineal scRNAseq (3). **b.** Dotplot of *Coch* expression as obtained from the reanalysis of published data (3) and integrating day time specific datasets of rat pineal scRNAseq.

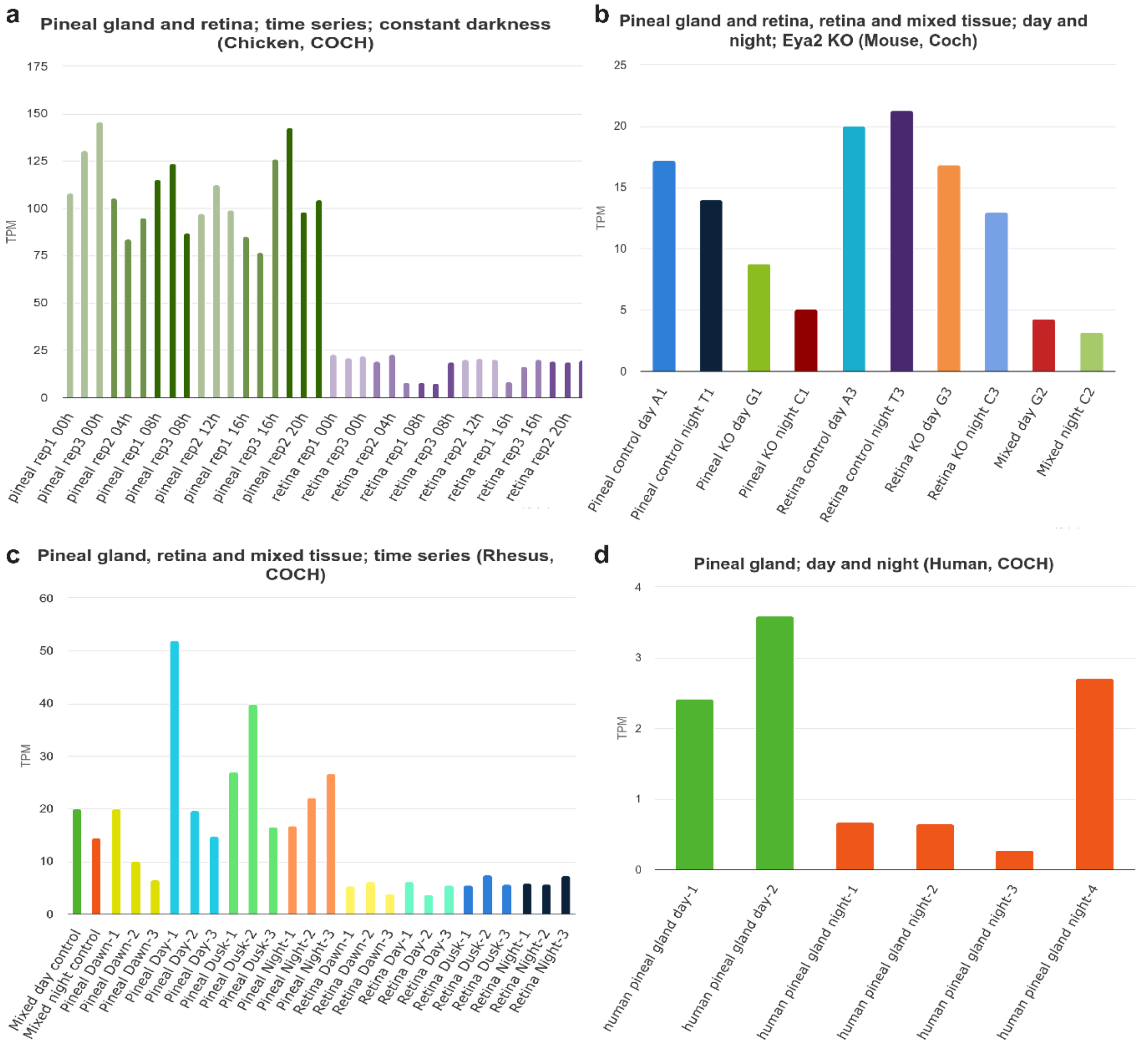

Figure S8. *Cochlin* expression in the pineal glands of other vertebrates. Data from the chicken (a), mouse (b) rhesus monkey (c) and humans (d) (4).

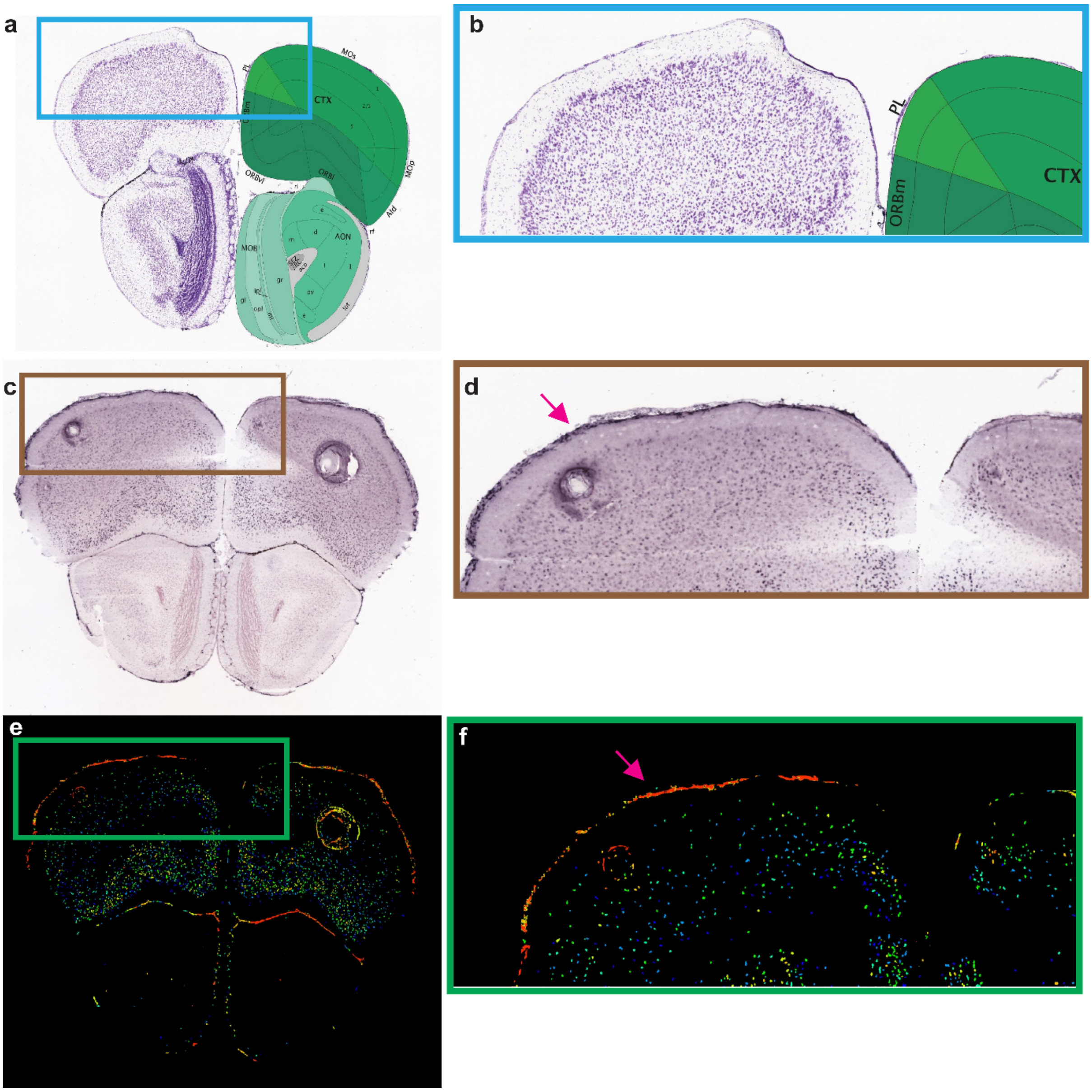

Figure S9: *Cochlin* expression in the adult mouse meninges. a. Nissl (left) and anatomical annotations (right) from the Allen Mouse Brain Atlas and Allen Reference Atlas – Mouse Brain, at the same region as (c) and (e). **b.** Anatomical annotations of zoomed out inset from panel a. Ctx - cortex, ORBm - Orbital area medial part, PL- Prelimbic area. Allen Reference Atlas – Mouse Brain (5). **c.** Expression of *Coch* in adult mouse brain (ISH). Allen Mouse Brain Atlas, mouse.brain-map.org/experiment/show/71717614 in the forebrain (5–8). **d.** Zoomed in inset from panel c. Magenta arrow: leptomeningeal staining. **e.** Expression heatmap of the section presented in panel c. **f.** Zoomed in inset from panel e. Magenta arrow: high expression in the leptomeninges.

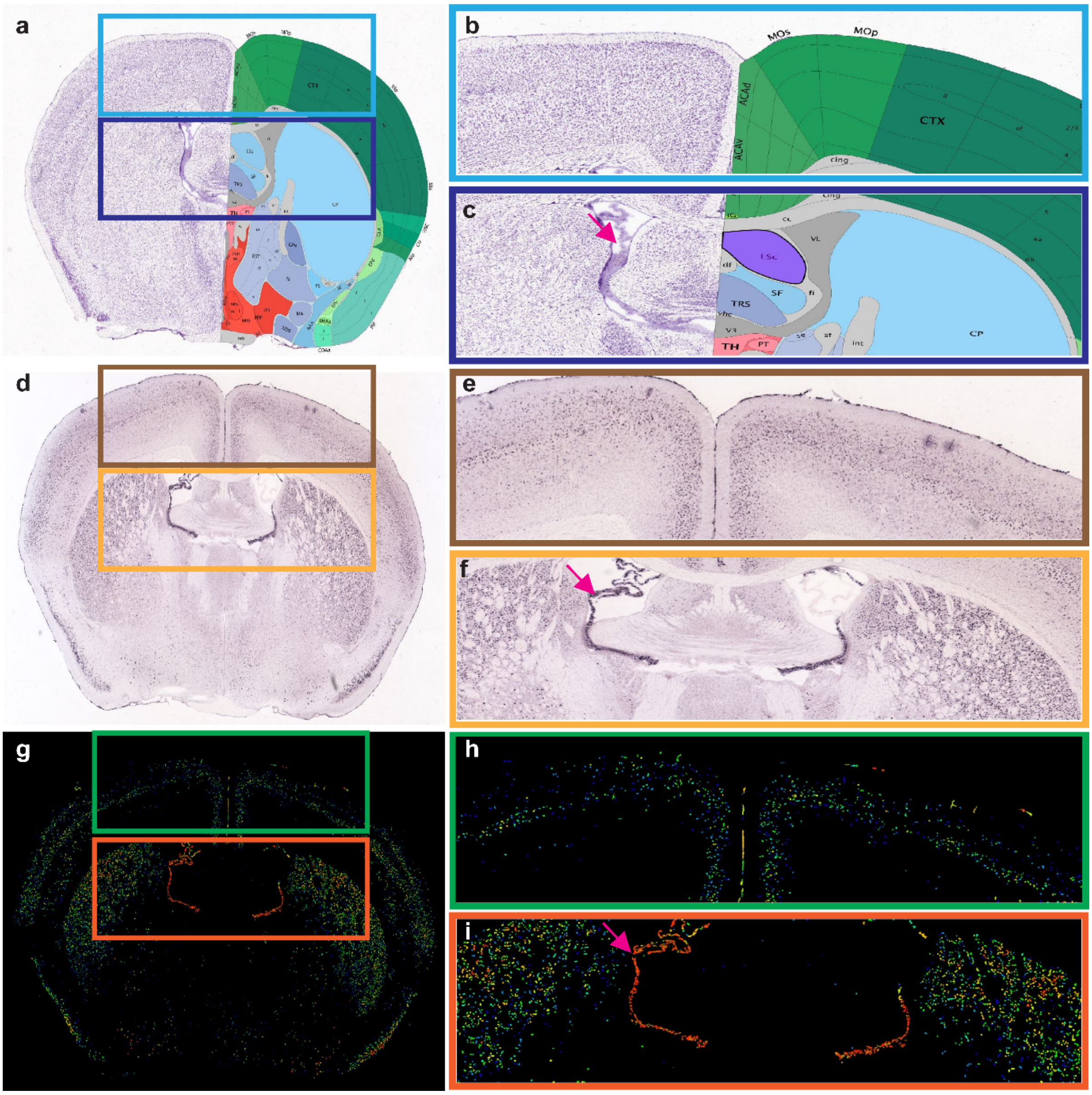

Figure S10: *Cochlin* expression in the adult mouse brain. a. Nissl (left) and anatomical annotations (right) from the Allen Mouse Brain Atlas and Allen Reference Atlas – Mouse Brain, at the same slice position as (d) and (g). **b, c.** Anatomical annotations of zoomed out inset from (a)- Ctx- cortex, ACAv- Anterior cingulate area -ventral, ACAd- Anterior cingulate area – dorsal, MOp- Primary motor area, Mos- Secondary motor area, df – dorsal fornix, VL – lateral ventricle, CP-Caudoputamen. Allen Reference Atlas – Mouse Brain (5). **d.** Expression of *Coch* in adult mouse brain (ISH). Allen Mouse Brain Atlas, mouse.brain-map.org/experiment/show/71717614 in the forebrain at the level of the septal nuclei (5–8). **e, f.** Zoomed in insets from panel d. **g.** Expression heatmap of the section presented in panel c. **h, i.** Zoomed in inset from panel e. Magenta arrows in panels f and i: high expression in the

choroid plexus of VL.

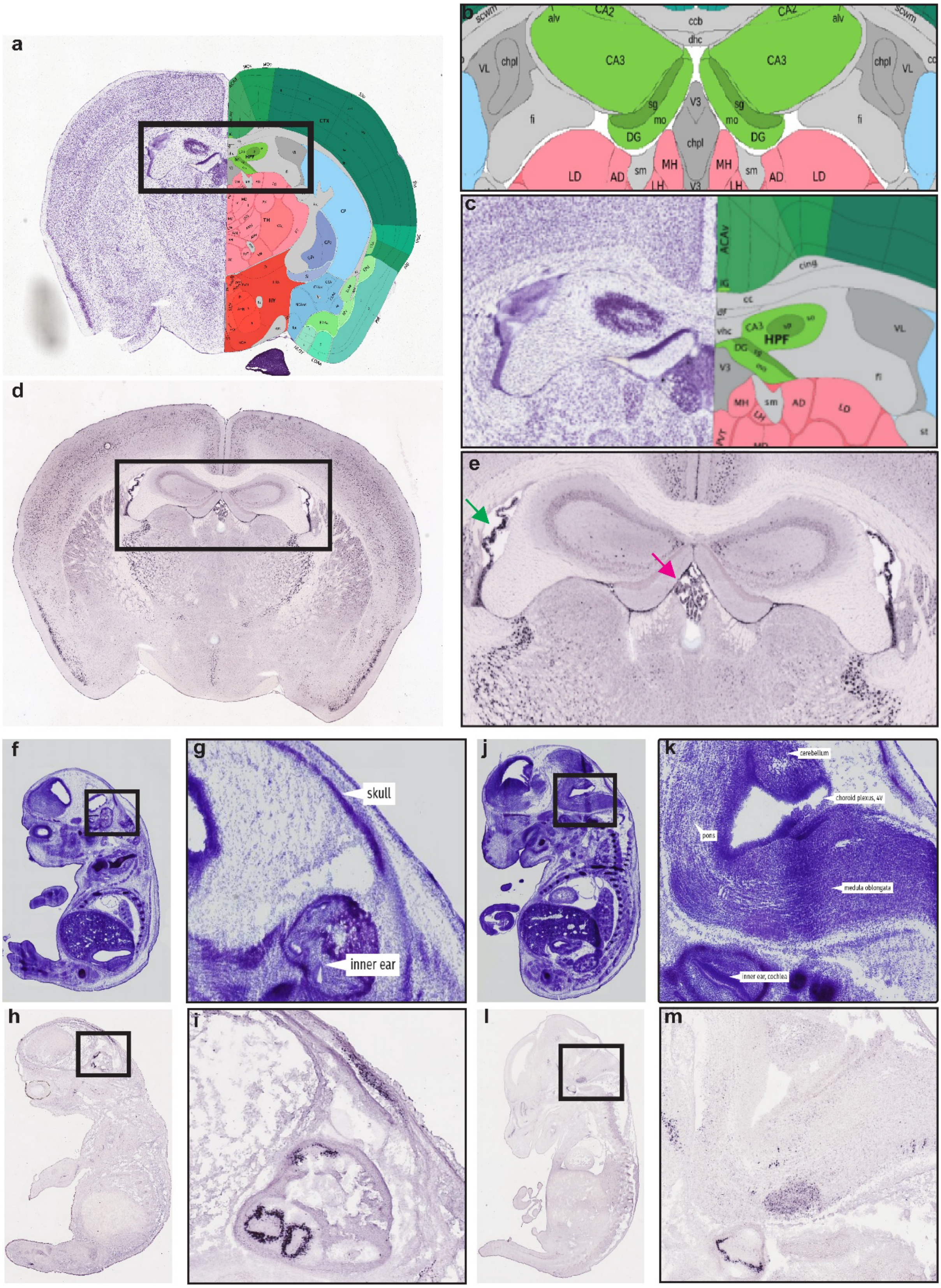

Figure S11: *Cochlin* expression in the choroid plexus. a. Nissl (left) and anatomical annotations (right) from the Allen Mouse Brain Atlas and Allen Reference Atlas – Mouse Brain, at the same slice position as panels b, c & d. **b.** Anatomical annotations of zoomed out inset from (a)- chpl-choroid plexus, VL-lateral ventricle, V3- third ventricle. Allen Reference Atlas – Mouse Brain (5). **c.** Nissl (left) and anatomical annotations(right) of zoomed in inset from panel a showing the structure of choroid plexus in the lateral and third ventricle. Allen Mouse Brain Atlas and Allen Reference Atlas – Mouse Brain (7). **d.** Expression of *Coch* in adult mouse brain (ISH). Allen Mouse Brain Atlas, mouse.brain-map.org/experiment/show/71717614 (5–8). **e.** Zoomed in inset from (d). green arrow- choroid plexus in the lateral ventricle, magenta arrow- choroid plexus of the third ventricle. **f-j.** Reference atlas for embryonic day 14.5 (E14.5) with Thioninacetate (Nissl-method) stain available on Genepaint (9). **g.** Zoomed in inset from (f) showing location of the inner ear and skull. **k.** Zoomed in inset from (j) showing location of the fourth ventricle (4V) choroid plexus. **h, l.** ISH for stained *Coch* in E14.5 mouse as available from https://gp3.mpg.de/results/Coch (9). **i.** Zoomed in inset from (h) showing staining of the inner ear, which serves as a positive control. **m.** Zoomed in inset from (l) showing the anatomical regions near the choroid plexus of the fourth ventricle.

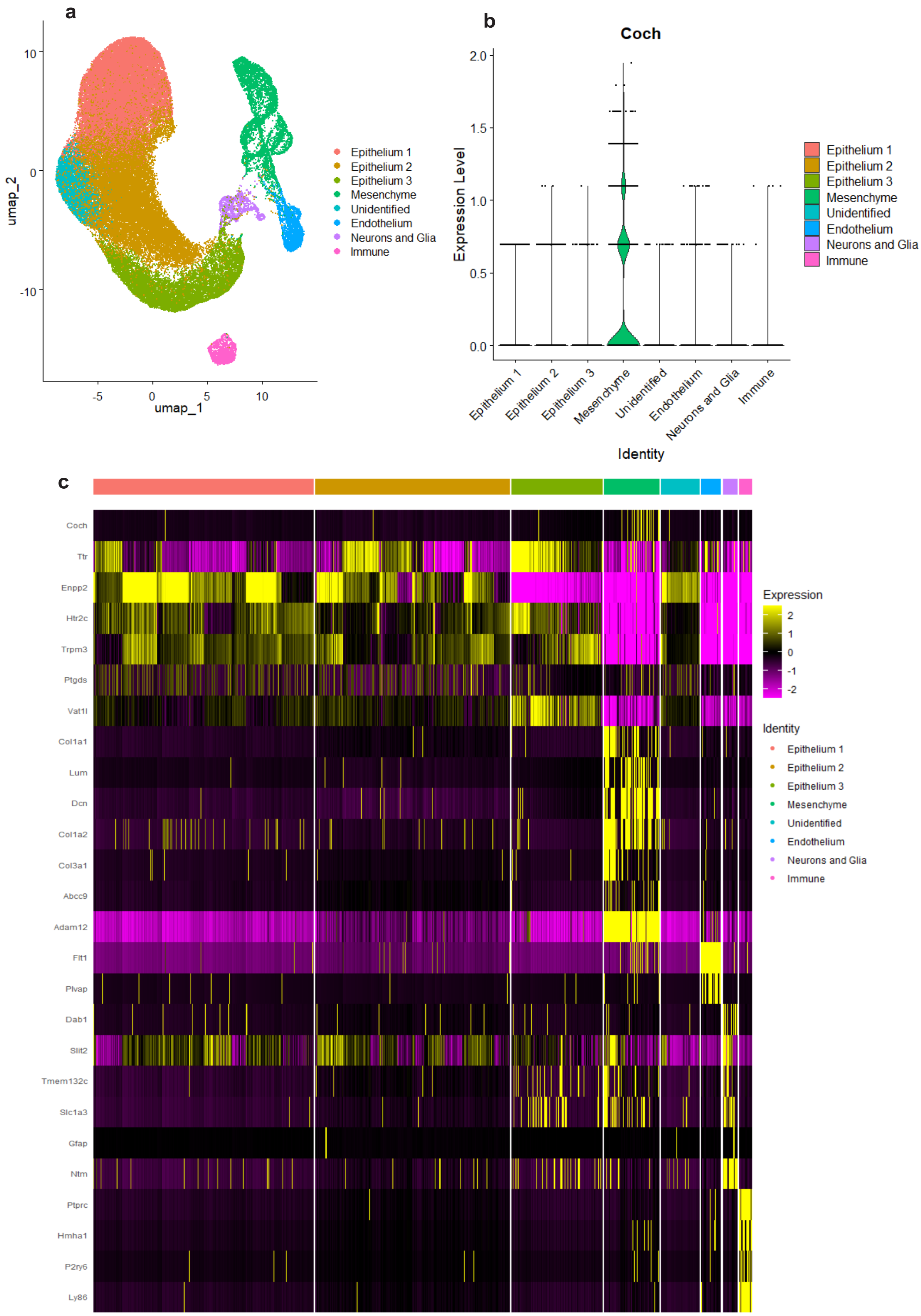

Figure S12. *Coch* expression in murine choroid plexus. a. UMAP of the reanalysed murine choroid plexus snRNA dataset (10). **b.** Violin plot of *Coch* expression as the clustering

described in (a). **c.** Heatmap with selected markers used for cluster annotation along with *Coch*.

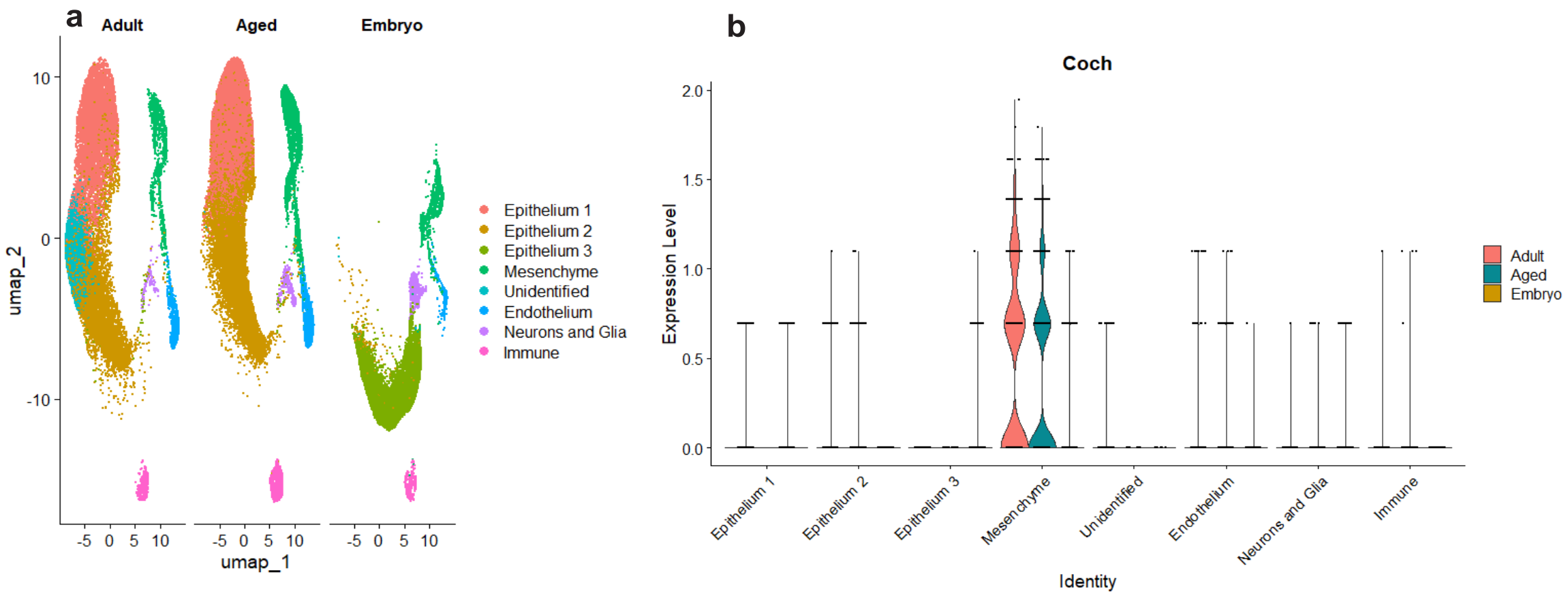

Figure S13. *Coch* expression the mouse choroid plexus at different ages. a. UMAP of the reanalysed murine choroid plexus snRNA dataset split by the age of the samples (10). **b.** Violin plot of *Coch* expression, split by age of the samples and clustered as described in Figure S9a.

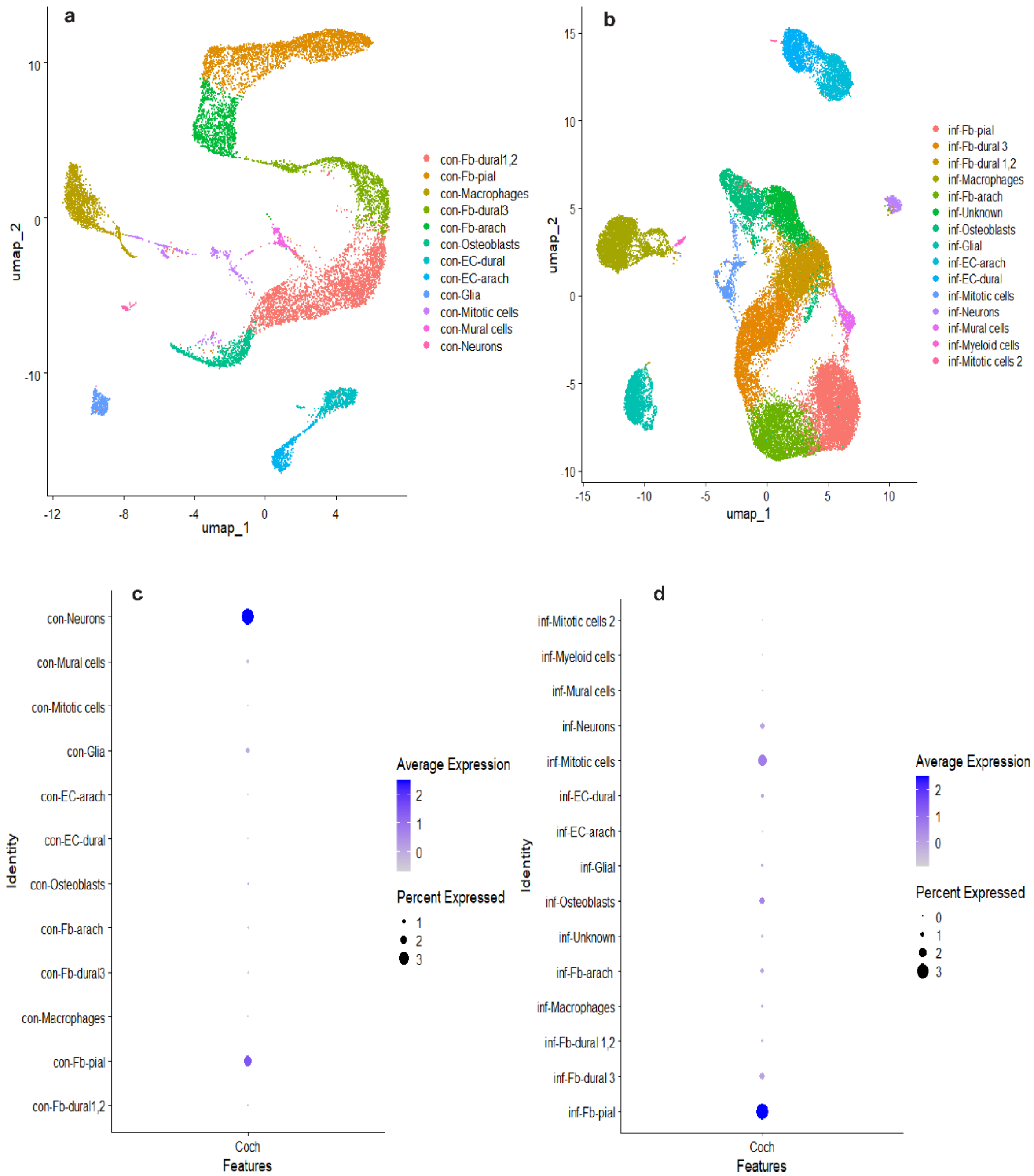

Figure S14. Murine meningeal fibroblast expression of *Coch* in early post-natal mice with and without *E.coli* meningitis . a-b. UMAPs of the integrated Seurat objects of meningeal cells of control (a) and infected (b) mice, based on reanalysis of GSE221678 (11). **c-d.** Dotplot for *Coch* expression in the integrated control (c) and infected datasets (d). con – control, inf- infected, Fb- fibroblasts, arach- arachnoid, EC- endothelial cells.

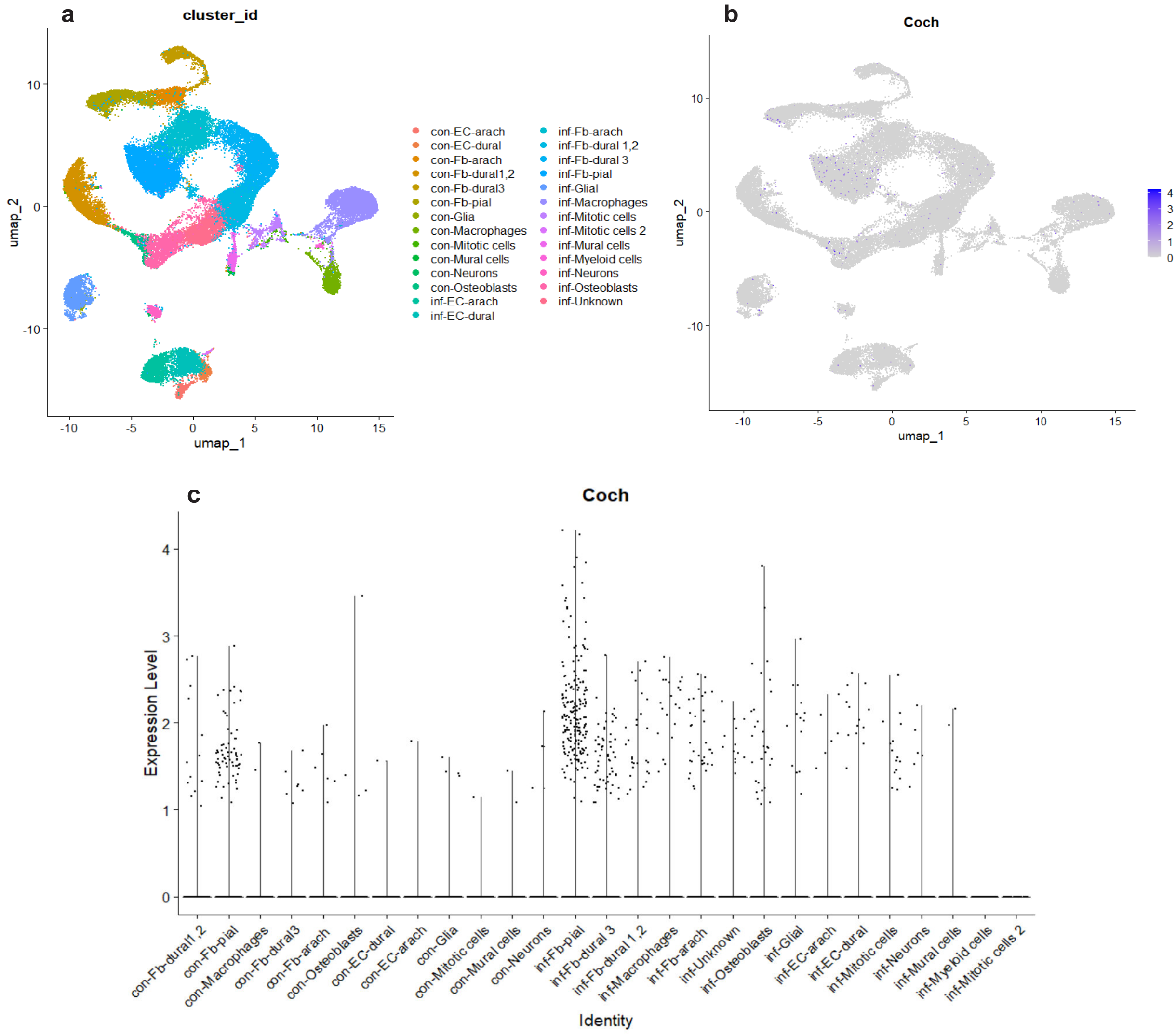

Figure S15. Murine meningeal expression of *Coch* increases in early post-natal mice with *E.coli* meningitis. a. UMAP of the merged Seurat object of meningeal cells of control and infected mice based on reanalysis of GSE221678 (11). **b.** Feature plot for *Coch* expression in the merged Seurat object. **c.** Violin plot for *Coch* expression in the merged Seurat object. con – control, inf- infected, Fb- fibroblasts, arach- arachnoid, EC- endothelial cells.

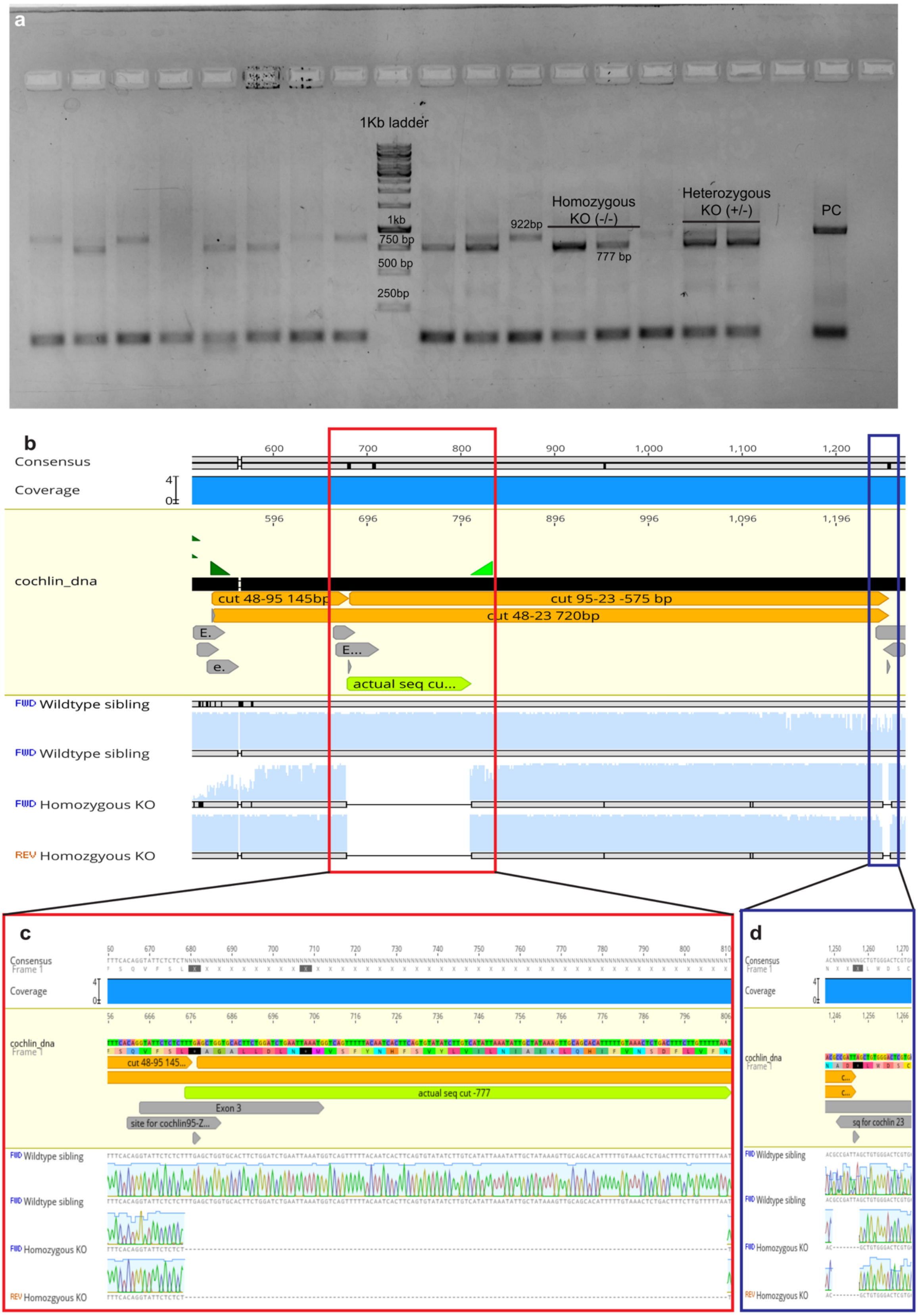
a
1Kb ladder
1kb
750 bp
500 bp
250bp
922bp
Homozygous KO (-/-)
777 bp
Heterozygous KO (+/-)
PC
b
Consensus
Coverage
cochlin_dna
cut 48-95 145bp
cut 95-23 -575 bp
cut 48-23 720bp
actual seq cu...
FWD Wildtype sibling
FWD Wildtype sibling
FWD Homozygous KO
REV Homozgyous KO
c
Consensus
Coverage
cochlin_dna
cut 48-95 145...
actual seq cut -777
Exon 3
site for cochlin95-Z...
FWD Wildtype sibling
FWD Wildtype sibling
FWD Homozygous KO
REV Homozgyous KO
d
Consensus
Coverage
cochlin_dna
sq for cochlin 23
FWD Wildtype sibling
FWD Wildtype sibling
FWD Homozygous KO
REV Homozgyous KO

Figure S16. Sequencing of the *cochlin* gene in *coch*[lkcsj1]. **a.** Gel electrophoresis image of genotyping PCR of offspring derived from in-cross of F1 *cochlin* heterozygous mutants. PC- Positive control. **b.** Overview of the sequencing results of two homozygous mutant (-/-) and one wt (+/+) sibling. **c.** Deletion at the cut site for cochlin95 gRNA. **d.** Deletion at the cut site for cochlin23 gRNA.

TableS1. Sex of adult HCR-ISH samples

Genes	Female	Male
rgra	*n* = 4 (Represented in the FigS11 q-t)	*n* = 2

Raw gel image (for Fig S16)

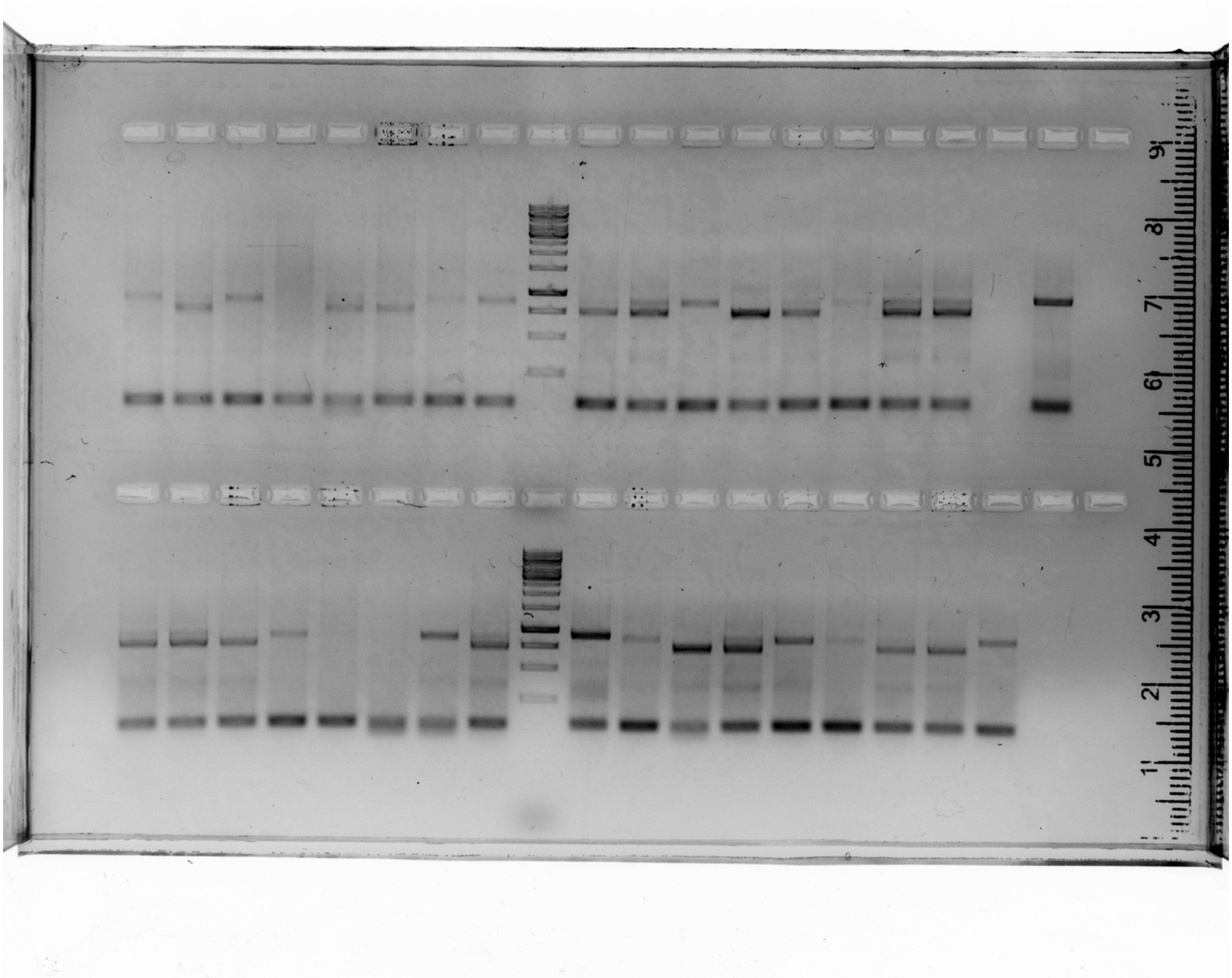

References

1. Elazary Y, Cheow K, Cheng R, Ghosh R, Shainer I, Wexler Y, et al. Glial cells expressing visual cycle genes are vital for photoreceptor survival in the zebrafish pineal gland. J Pineal Res. 2023 Apr;74(3). doi:10.1111/jpi.12854

2. Galanternik MV, Castranova D, Gober RD, Nguyen T, Kenton M, Margolin G, et al. Anatomical and Molecular Characterization of the Zebrafish Meninges. BioRxiv Prepr Serv Biol. 2025 Apr 15;2025.04.09.646894. doi:10.1101/2025.04.09.646894 PubMed PMID: 40321204; PubMed Central PMCID: PMC12047863.

3. Mays JC, Kelly MC, Coon SL, Holtzclaw L, Rath MF, Kelley MW, et al. Single-cell RNA sequencing of the mammalian pineal gland identifies two pinealocyte subtypes and cell type-specific daily patterns of gene expression. Yamazaki S, editor. PLOS ONE. 2018 Oct 22;13(10):e0205883. doi:10.1371/journal.pone.0205883

4. Chang E, Fu C, Coon SL, Alon S, Bozinoski M, Breymaier M, et al. Resource: A multi-species multi-timepoint transcriptome database and webpage for the pineal gland and retina. J Pineal Res. 2020 Oct;69(3):e12673. doi:10.1111/jpi.12673

5. Reference Atlas :: Allen Brain Atlas: Mouse Brain [Internet]. [cited 2024 Jul 31]. Available from: https://mouse.brain-map.org/static/atlas

6. Daigle TL, Madisen L, Hage TA, Valley MT, Knoblich U, Larsen RS, et al. A Suite of Transgenic Driver and Reporter Mouse Lines with Enhanced Brain-Cell-Type Targeting and Functionality. Cell. 2018 Jul;174(2):465-480.e22. doi:10.1016/j.cell.2018.06.035

7. ISH Data :: Allen Brain Atlas: Mouse Brain [Internet]. [cited 2024 Jul 31]. Available from: https://mouse.brain-map.org/

8. Lein ES, Hawrylycz MJ, Ao N, Ayres M, Bensinger A, Bernard A, et al. Genome-wide atlas of gene expression in the adult mouse brain. Nature. 2007 Jan;445(7124):168–76. doi:10.1038/nature05453

9. Genepaint - Home of High Resolution Gene Expression Data [Internet]. [cited 2024 Jul 31]. Available from: https://gp3.mpg.de/

10. Dani N, Herbst RH, McCabe C, Green GS, Kaiser K, Head JP, et al. A cellular and spatial map of the choroid plexus across brain ventricles and ages. Cell. 2021 May;184(11):3056-3074.e21. doi:10.1016/j.cell.2021.04.003

11. Wang J, Rattner A, Nathans J. Bacterial meningitis in the early postnatal mouse studied at single-cell resolution. eLife. 2023 Jun 15;12:e86130. doi:10.7554/eLife.86130
